# 3.0 T multi-parametric MRI reveals metabolic and microstructural abnormalities in the posterior visual pathways in patients with thyroid eye disease

**DOI:** 10.3389/fnins.2023.1306364

**Published:** 2024-01-09

**Authors:** Lan Luo, Liling Zhang, Huaidong Huang, Jitian Guan, Xiaolei Zhang, Yan Lin, Renhua Wu

**Affiliations:** ^1^Radiology Department, Second Affiliated Hospital of Shantou University Medical College, Shantou, Guangdong Province, China; ^2^Radiology Department, Huizhou Central People’s Hospital, Huizhou, Guangdong Province, China; ^3^Endocrinology Department, Second Affiliated Hospital of Shantou University Medical College, Shantou, Guangdong Province, China

**Keywords:** visual pathways, thyroid eye disease, magnetic resonance spectroscopy, glutamate chemical exchange saturation transfer, diffusion kurtosis imaging

## Abstract

**Introduction:**

We aim to explore the microstructural and metabolic changes in visual pathways in patients with thyroid eye disease (TED) using 3T multi-parametric MRI.

**Methods:**

Thirty-four TED patients (inactive group = 20; active group = 14; acute group = 18; chronic group = 16) and 12 healthy controls (HC) were recruited from November 2020 to July 2021. Proton magnetic resonance spectroscopy (^1^H-MRS), glutamate chemical exchange saturation transfer (GluCEST) and diffusion kurtosis imaging (DKI) were performed on 3.0T MR scanner. Data analysis and group comparisons were performed after MR data processing.

**Results:**

As compare to HC group, the levels of total choline (tCh) in optic radiation (OR) in active group ([1.404 ± 0.560] vs. [1.022 ± 0.260]; *p* < 0.05), together with tCh ([1.415 ± 0.507] vs. [1.022 ± 0.260]; *p* < 0.05) in OR in acute group were significantly increased. Glutamine (Gln) levels were higher in OR in the chronic group than those in HCs and were positively correlated with the levels of thyroglobulin antibody (TgAb), thyroid peroxidase antibody (TPOAb), free triiodothyronine (FT3) and FT4 in chronic group. Glutamate (Glu) levels by ^1^H-MRS did not show significant differences between any two groups. Interestingly, MTRasym (3.0 ppm) was higher in OL in inactive group, active group, acute group and chronic group than those in HCs, and was positively correlated with Glu levels in OL in ^1^H-MRS. Fractional anisotropy (FA) values from DKI in OR in acute group were significantly lower than those in HCs.

**Discussion:**

Our initial study demonstrate that GluCEST performs better than ^1^H-MRS to monitor Glu alterations in visual pathway in TED patients. Changes of brain glutamine levels in TED patients are closely related to their associated hormones alterations, indicating that disease injury status could be reflected through non-invasive metabolites detection by brain ^1^H-MRS. FA is the most sensitive DKI index to reveal the visual pathway impairment in TED patients. Altogether, our study revealed that 3T multiparametric MR techniques are useful to demonstrate metabolic and microstructural alterations in visual pathways in TED patients. We found that damage to visual pathways occurs in mild TED cases, which not only offers a new approach to the diagnosis of dysthyroid optic neuropathy, but also demonstrates neuropathy in TED is a gradual and continuous spatio-emporal progression.

## Introduction

Thyroid eye disease (TED), also known as thyroid-associated ophthalmopathy (TAO) or Graves’ orbitopathy, is a complex autoimmune disease that mainly presents with ocular changes, leading to a significant reduction in patients’ quality of life and psychological health, with the worst cases showing dysthyroid optic neuropathy (DON) (Riguetto et al., 2019; Taylor et al., 2020). Pathological evaluations of TED patients include extraocular muscle destruction, various pro-inflammatory factors and lymphocyte infiltration (Smith and Hegedüs, 2016). Many previous studies based on optical coherence tomography and 3 T brain magnetic resonance imaging (MRI) showed that damage to the optic nerve could be transmitted along the visual pathway to the visual radiation and the occipital lobe (Tur et al., 2016). Currently, by the use of Ultrasound, ophthalmoscope and optical coherence tomography techniques, clinical visual detection is subjective and lacks precision to assess the onset of DON (Zhang et al., 2019; Ji et al., 2021; Zhang et al., 2021). Therefore, it is essential to find a simple, non-invasive and effective technique to assess the status of intracranial visual pathway in TED patients.

More and more people show interest in DON research, for visual impairment in thyroid-related eye disease is a critical event that affects treatment options (Garip-Kuebler et al., 2021; Ji et al., 2021). However, the diagnosis of DON is mainly through abnormal visual function and gross anatomy. We do not exactly know when the optic nerve injury occurs. At present, conventional MRI diagnosis of TED is mainly focused on the observation of the extraocular muscle hypertrophy and optic nerve stretching (Rutkowska-Hinc et al., 2018), which is limited to the anatomical changes of intraorbital lesions. Diffusion tensor imaging (DTI) has been performed to study microstructural changes of the optic nerve in severe TED patients (Lee et al., 2018; Chen et al., 2020), which indicated that MD (mean diffusivity), AD (axial diffusivity) and RD (radial diffusivity) were significantly lower in TED patients than those in healthy controls. Diffusion kurtosis imaging (DKI), as a direct extension of the DTI model for a non-Gaussian distribution (Xu et al., 2018; Li et al., 2020), has been shown to better characterize the microstructural alterations of the brain, but recently there are no relevant reports of DKI in TED patients. *In vivo* proton MR Spectroscopy (^1^H-MRS) provides a simple, efficient and inexpensive technical support for detecting the molecular and biochemical information of the brain. Elberling and Danielsen et al. has studied the metabolic changes by ^1^H-MRS in parietal-occipital lobe in patients with acute thyrotoxic graves disease before and after treatment, and found that glutamine levels were consistently significantly reduced in parietal-occipital lobe (Elberling et al., 2003). The glutamate (Glu) levels can be measured by ^1^H-MRS and chemical exchange saturation transfer (CEST), the latter is a relatively new and promising MRI contrast technique which can efficiently investigate changes in proteins, neurotransmitters and other metabolites (Mehrabian et al., 2017). More importantly, GluCEST can produce high-resolution images covering large areas of the brain (Sydnor et al., 2021), compared to traditional ^1^H-MRS. So far, there are no reports on GluCEST to study the changes of exchange rate between Glu and water in the visual pathway with TED involving the intraorbital region.

Given that it is not clear whether there are metabolic and microstructural alterations in intracranial visual pathway in TED patients, and multi-parametric MRI has advantages in diagnosing and assessing intracranial changes compared with any other radiological technique. Hence, this study aimed to explore the metabolic and microstructural changes in visual pathways in TED patients by using ^1^H-MRS, GluCEST and DKI, which were expected to provide objective references for TED patients before the onset of disabling DON.

## Materials and methods

### Participants

The study was approved by the Ethics Committee. Informed consent was obtained from the TED patients before MRI examinations. Thirty-four patients underwent magnetic resonance scans from November 2020 to July 2021. A clinical diagnosis of TED was made if the examination showed that thyroid dysfunction or dysregulation was objectively correlated with ocular symptoms. Ocular symptoms include minor lid retraction (<2 mm), mild soft-tissue involvement, exophthalmos <3 mm above normal for ethnicity and sex, no or intermittent diplopia, and corneal exposure responsive to lubricants. Patients were eligible for inclusion in this study if they were 18 to 70 years of age. The exclusion criteria were as follows: (1) mental illness; (2) other neurological conditions, including brain tumor, cerebral infarction or hemorrhage, brain trauma, and degeneration injury; (3) other conditions that may cause unilateral or bilateral exophthalmos or extraocular muscle enlargement, such as Cushing’s syndrome, orbital pseudotumor, idiopathic myositis, and cellulitis; (4) visual loss. 16 cases are in the exclusion criteria. Activity measures were based on the following classic features of inflammation constituting the clinical activity score (CAS): (1) spontaneous retrobulbar pain; (2) pain on attempted up, down, or side gaze; (3) redness of the eyelids; (4) redness of the conjunctiva; (5) swelling of the eyelids; (6) swelling of the caruncle, plica, or both; (7) chemosis and the presence of more than three features indicating active TED. Acute and chronic conditions were determined based on whether the disease duration was >6 months (inactive group = 20; active group = 14; acute group = 18; chronic group = 16). Healthy age- and sex-matched controls (HCs, n = 12) were recruited.

### Magnetic resonance imaging

#### Magnetic resonance spectroscopy

**Figure 1:**
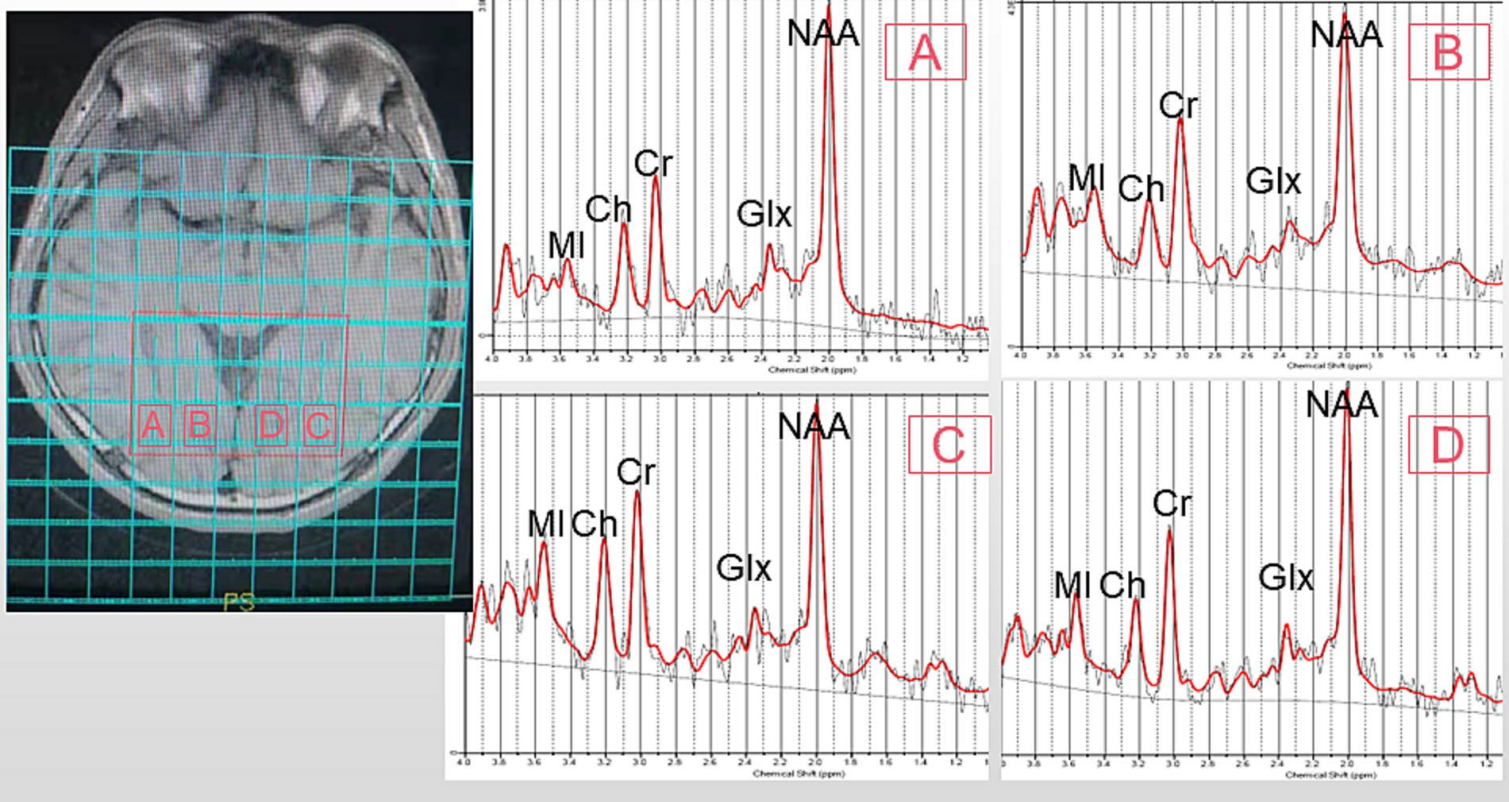
Representative MRS spectrum. The ROI is shown in an axial T1 fluid-attenuated inversion recovery (T1FLAIR) image. **(A)**: right optic radiation (ROR), **(B)**: right occipital lobe (ROL), **(C)**: left optic radiation (LOR), **(D)**: left occipital lobe (LOL). MI, MyoInositol; Ch, Choline-containing Compounds; Cr, Creatine; NAA, N-Acetylaspartate; Glx, Glutamate + Glutamine.

All participants underwent T1 fluid-attenuated inversion recovery (T1-FLAIR) imaging and ^1^H-MRS using a 3 T General Electric MR Scanner (Sigma; GE Healthcare, Milwaukee, WI, USA). T1-FLAIR images acquired with the following parameters: repetition time [TR], 1750 ms; echo time [TE], 24 ms; inversion time [TI], 780 ms; matrix, 320 × 224; and field of view [FOV], 24 × 24 cm, were used for anatomical localization. Multivoxel spectral data were obtained at 8 min and 12 s upon applying water-suppressed point-resolved spectroscopy (PRESS) pulse sequence (TR, 3500 ms; TE, 29 ms; FOV, 16 cm × 16 cm; matrix size, 11 × 11; slice thickness, 5 mm; FWHM<20hz; number of repeated acquisitions = 1; water suppression method: Chemical shift selective (CHESS) pulse; voxel size = 7.5*4 cm). The entire voxel was located in the OR and the occipital cortex of the brain, shown in Figure 1. Data post-processing and quantification were analyzed using LCModel (version 6.3). The Cramer-Rao lower bounds of all metabolite to be analyzed <15%. On axial T1-FLAIR images, a line was made between the zygomatic process on both sides, and the vertical distance between the front of the eyeball and the line was measured, which is exophthalmic extent. Blood tests were taken three days before or after the MRI.

#### GluCEST

**Figure 2:**
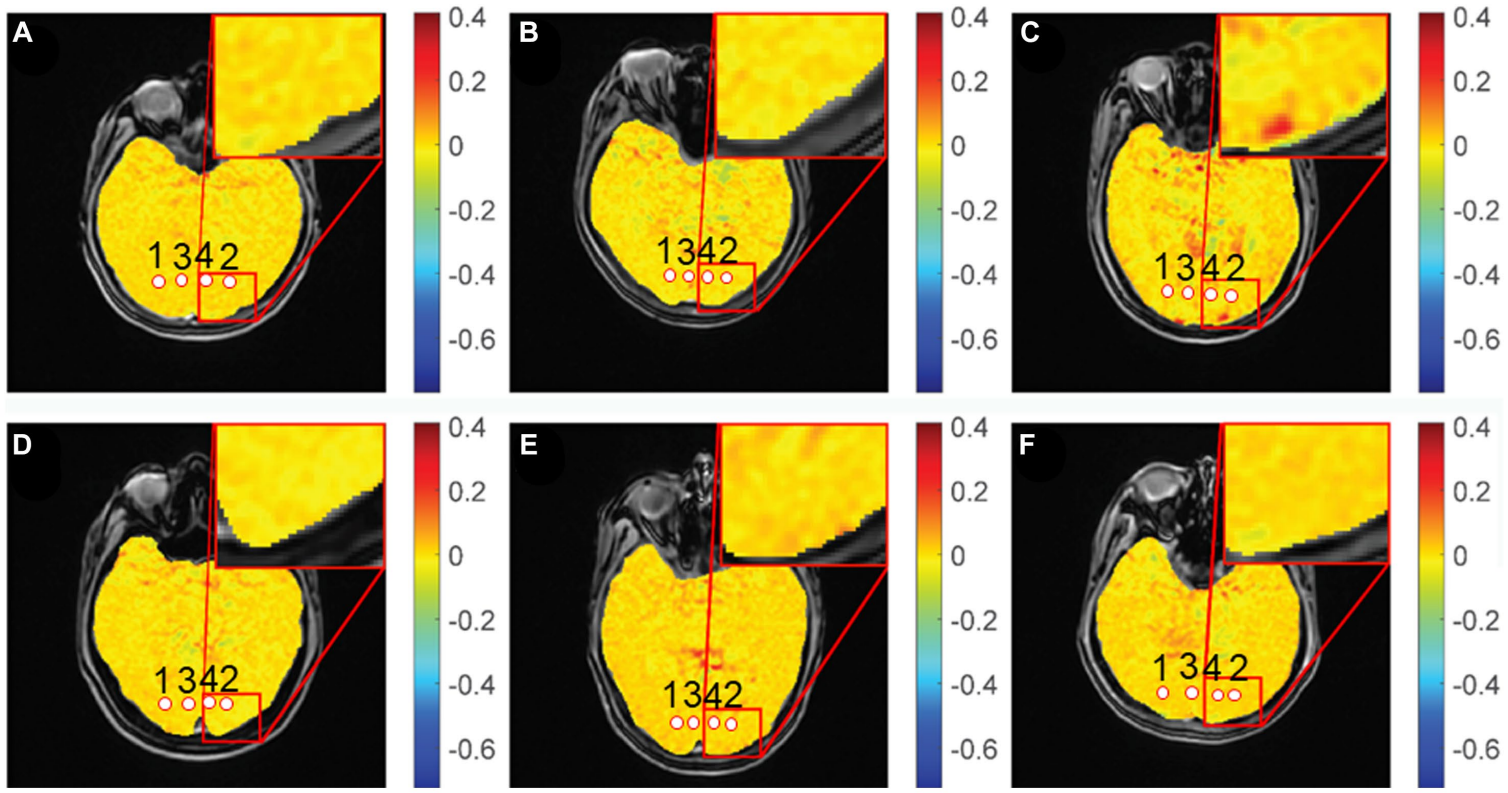
GluCEST% maps of different groups. **(A,D)**: healthy control, **(B)**: chronic group, **(C)**: acute group, **(E)**: inactive group, **(F)**: active group. 1, 2: optic radiation (OR), 3, 4: occipital lobe (OL).

Gradient echo MRI sequence with magnetization transfer (MT) was used for CEST imaging (TR, 50 ms; TE, 3.1 ms; FOV, 24 cm × 24 cm; matrix, 128 × 128; slice thickness, 5 mm; bandwidth, 15.63 kHz; flip angle, 60°). The MT saturation pulse was a Fermi pulse with a width of 4 ms and B1 of 0.2 μT. The scan yielded 2 equidistant frequency shifts (−3 to 3 ppm) and an extra S0 image. Image processing and data analysis were performed using MATLAB custom scripts (The Mathworks, Inc., Natick, MA, United States) (Mao et al., 2019; Tang et al., 2020). In the previous study, the Z-spectra show that the amine protons on Glu generated a CEST effect with a chemical shift of 3.0 ppm away from bulk water, and indicate that the GluCEST signal was concentration-dependent (Jia et al., 2020). Figure 2 showed the GluCEST contrast plot, also known as the magnetization transfer ratio (MTRasym). MTRasymcalculated as,

MTRasym = [S (−3 ppm) – S (+3 ppm)]/S0.

where S (−3 ppm) and S (+3 ppm) were the water signals of the saturation pulse at an offset of ±3 ppm against the water resonance, respectively, and S0 was the signal for water without a saturated pulse.

#### Diffusion kurtosis imaging

**Figure 3:**
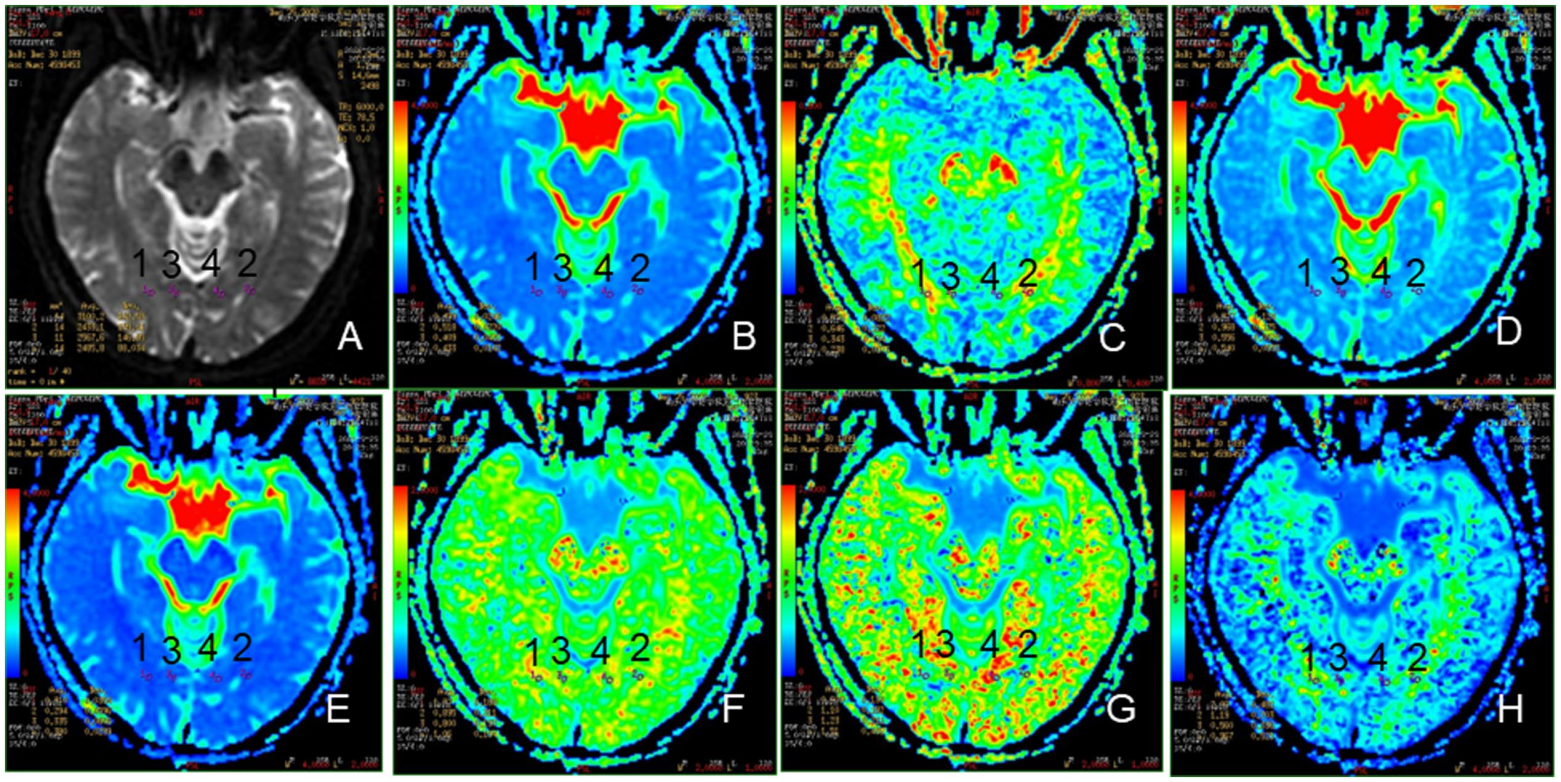
Representative region of interest (ROI) for DKI parameters. **(A)**: The raw map of DKI, **(B)**: MD map, **(C)**: FA map, **(D)**: Da map, **(E)**: Dr. map, **(F)**: MK map, **(G)**: Ka map, **(H)**: Kr map, 1, 2: optic radiation (OR), 3, 4: occipital lobe (OL).

The DKI scan was obtained using echo-planar imaging sequence (TR, 6000 ms; TE, 73.4 ms; FOV, 24 cm × 24 cm; matrix, 224 × 384; thickness, 5.0 mm; interslice gap, 5.0 mm; a number of slices, 20). Three b-values (0, 1,000, and 2,000 s/mm^2^) were applied in 15 directions, with a total acquisition time of 3 min and 5 s (Guan et al., 2019). The raw data (DICOM format) was sent to commercial workstations (GE, ADW 45, Rue de la Miniere, France) equipped with Functool software. The DKI and T1-FLAIR images were opened at the same time on the workstations, and after double magnification, the area of interest is drawn on the T1-FLAIR image at the chiasmatic - optic radiation-occipital lobe level by a neuro-radiologists after one-year training to obtain mean diffusivity (MD), axial diffusivity (Da), radial diffusivity (Dr), fractional anisotropy (FA), mean kurtosis (MK), axial kurtosis (Ka), and radial kurtosis (Kr) parameter maps. The regions of interest in the OR and occipital cortex were independently drawn as a fixed-size (10–20 mm^2^) ellipse, as shown in Figure 3.

#### Statistical analysis

All data were analyzed using SPSS 25 statistical software (version 24, IBM Corp., Armonk, NY, United States). Normality test and variance homogeneity test were performed first for the three groups of data (^1^H-MRS, DKI parameters, GluCEST and age). If the three groups of data conform to normal distribution, One-way Analysis of Variance (ANOVA) was used to compare the differences of the three group. LSD (least significant difference) test was used for comparison between groups. If the variance of individual parameters was uneven or did not conform to normal distribution, Kruskal-Wallis rank sum test was applied to analyze the differences between groups. Chi-square (χ^2^) tests were used to compare sex between the TED groups and HCs. Two-sided *p*-values less than 0.05 indicated statistical significance. The relationships between metabolite concentrations and serum indices were analyzed using Spearman’s correlation coefficient. All numerical data were averaged and reported as mean ± standard deviation.

## Results

### Demographics and clinical characteristics

Compared with HCs, significant differences in proptosis were seen in both active TED groupand chronic TED group, but not in inactive and acute TED group. There were no significant differences in age and sex between the active, inactive, and HC group and between the acute, chronic, and HC group. In addition, no significant differences were seen in FT3, FT4, TSH, TRAb, TPOAb and TgAb between inactive and active group, and between chronic and acute group. All study subjects did not present with disabling dysthyroid optic neuropathy confirmed by clinical evaluation. Table 1 showed the patients’ demographics and clinical characteristics.

**Table 1:** Clinical features and demographic information between TED groups and HCs.

		Inactive group	Active group	Chronic group	Acute group	Healthy control
Age (years)		39 ± 12	44 ± 12	39 ± 10	41 ± 14	41 ± 14
Sex (female/male)		11/9	6/8	8/10	9/7	9/3
TRAb (IU/L)		2.65(0.25–199)	10.7(2.16–28.61)	8.97(0.25–199)	3.76(0.25–28.61)	(ref. range, <1.5 IU/L)
TPOAb (IU/mL)		23.1(15–500)	40(15–500)	44(15–500)	25.35(15–500)	(ref. range, <60 IU/mL)
TgAb (IU/mL)		41.4(28–1,300)	1,300(28–1,300)	66.9(28–1,300)	379.2(28–1,300)	(ref. range, <60 IU/mL)
FT3 (pmol/L)		5.46(2.45–11.25)	5.72(2.94–32.17)	5.46(2.45–11.48)	5.57(3.7–32.17)	(ref. range, 3.28–6.47 pmol/L)
FT4 (pmol/L)		12.83(6.08–36.8)	12.12(4.55–54.66)	12.57(6.24–32.41)	12.4(4.55–54.66)	(ref. range, 7.64–16.03 pmol/L)
TSH (mIU/L)		0.23(0.01–35.02)	0.06(0.01–49)	0.15(0.01–4.75)	0.023(0.01–49)	(ref. range, 0.49–4.91 mIU/L)
Proptosis, (mm)	Left	18.88 ± 2.83^α^	22.33 ± 2.63^αα^	21.31 ± 3.83^αα^	19.36 ± 2.06^α^	16.51 ± 3.26
	Right	18.60 ± 2.84	22.05 ± 2.89^αα^	21.04 ± 3.70^αα^	19.05 ± 2.56	16.53 ± 3.96

α: statistical difference in ANOVA between the three groups (Inactive group, Active group and Healthy control, or Chronic group, Acute group, and Healthy control); compared with the control group, α: *p* < 0.05; αα: *p* < 0.01.

### Metabolic alterations in TED patients by ^1^H-MRS

As can be seen in Table 2, the levels of total choline (tCh) in OR in active group were significantly higher, compared with HCs ([1.404 ± 0.560] vs. [1.022 ± 0.260]; *p* < 0.05) (The unit of metabolites in MRS is in an arbitrary unit). Compared with HC group, the amounts of glutamine (Gln) ([3.014 ± 2.929] vs. [1.035 ± 1.043]) in OR in chronic group, together with those of tCh ([1.415 ± 0.507] vs. [1.022 ± 0.260]) in OR in acute group were significantly higher (*p* < 0.05). There were no significant differences in Glu levels between any two groups, but a tendency increase was shown in TED groups compared to HCs.

**Table 2:** Value of FA in the different groups and locations.

	Healthy control	Inactive group	Active group	Chronic group	Acute group
RON	0.684 ± 0.094	0.681 ± 0.100	0.612 ± 0.081	0.670 ± 0.093	0.630 ± 0.102
LON	0.672 ± 0.092	0.635 ± 0.142	0.648 ± 0.084	0.659 ± 0.125	0.617 ± 0.112
ROR	0.533 ± 0.093	0.480 ± 0.106	0.493 ± 0.085	0.525 ± 0.085^α^	0.438 ± 0.091^α^
LOR	0.479 ± 0.105	0.468 ± 0.057	0.454 ± 0.063	0.479 ± 0.056	0.441 ± 0.058
ROL	0.326 ± 0.097	0.268 ± 0.087	0.277 ± 0.093	0.281 ± 0.091	0.261 ± 0.086
LOL	0.299 ± 0.101	0.289 ± 0.107	0.284 ± 0.108	0.333 ± 0.111^α^	0.231 ± 0.067^α^

RON: right optic nerve, LON: left optic nerve, ROR: right optic radiation, LOR: left optic radiation, ROL: right occipital lobe, LOL: left occipital lobe. α: statistical difference in ANOVA between the three groups (Inactive group, Active group and Healthy control, or Chronic group, Acute group, and Healthy control); compared with the control group, α: *p* < 0.05; αα: *p* < 0.01.

### Metabolic alterations in TED patients by GluCEST

**Figure 4:**
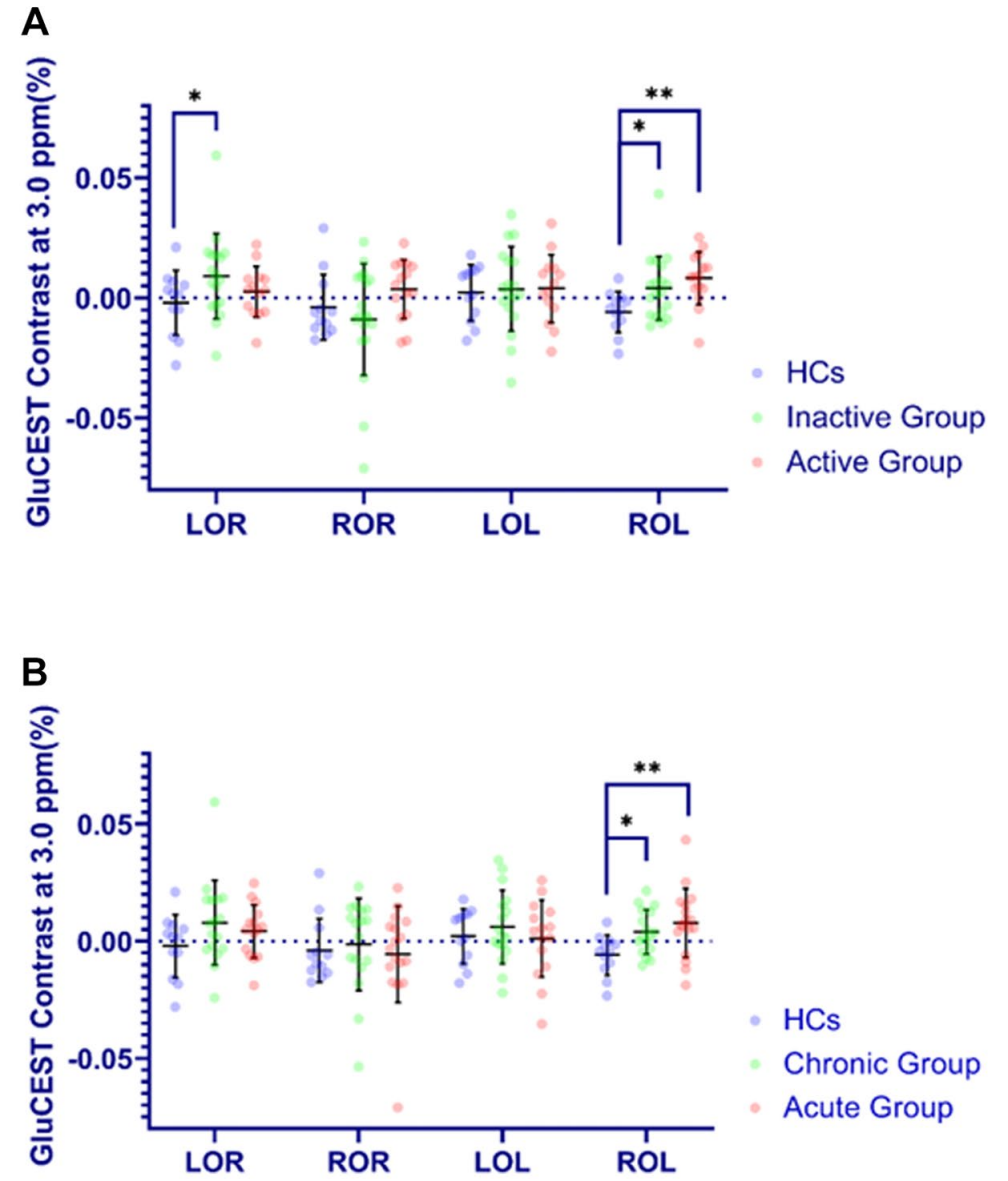
Comparison of GluCEST% contrast in four brain regions in different groups. The Y-axis (GluCEST contrast at 3.0 ppm %) uses raw data. **(A)** (Inactive or Active) vs. HC. **(B)** (Acute or Chronic) vs. HC. *p < 0.05, **p < 0.01.

As was shown in Figure 4, the GluCEST effect in OR in inactive group relative to HCs ([0.008 ± 0.023] vs. [−0.002 ± 0.013]) were significantly higher (p < 0.05). Meanwhile, the GluCEST effect in OL in inactive, active, chronic and acute groups as compared to HCs ([0.002 ± 0.010] vs. [−0.006 ± 0.009], [0.007 ± 0.013] vs. [−0.006 ± 0.009], [0.002 ± 0.010] vs. [−0.006 ± 0.009], [0.007 ± 0.013] vs. [−0.006 ± 0.009]; respectively) were also significantly increased (p < 0.05).

### Microstructural alterations in TED patients by DKI

In the optic nerve, the values of Ka ([0.702 ± 0.168]) in active group was significantly lower than those in HCs ([0.834 ± 0.151]), while Kr in both active and chronic group ([1.541 ± 0.528], [1.495 ± 0.659], respectively) were significantly higher compared with those in HCs ([1.092 ± 0.374]). As comparison with HCs, FA ([0.438 ± 0.091] vs. [0.533 ± 0.093]) and Da ([0.701 ± 0.092] (um^2^/ms) vs. [0.789 ± 0.079]) (um^2^/ms) in OR in acute group were lower, and MD ([0.469 ± 0.061] (um^2^/ms)vs. [0.422 ± 0.042] (um^2^/ms)) and Dr. ([0.410 ± 0.068] (um^2^/ms)vs. [0.352 ± 0.045] (um^2^/ms)) in OL were higher.

### Correlation analysis of metabolites with GluCEST and Grave’s disease (GD) associated hormones

**Figure 5:**
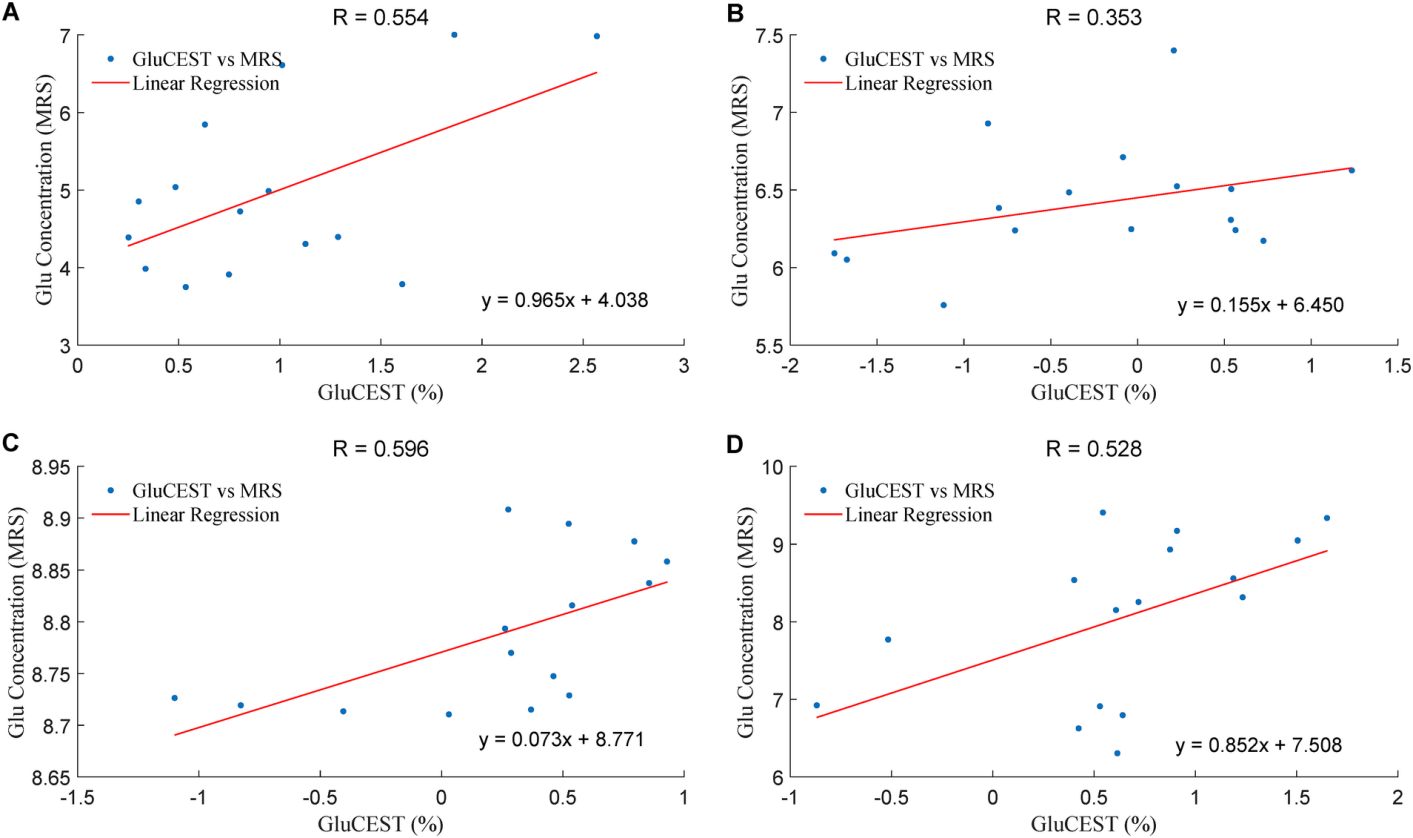
The correlation of the Glu levels in GluCEST and MRS in the acute group. The unit of Glu concentration (MRS) is in an arbitrary unit, the X-axis (GluCEST %) is raw data ×100%. **(A)**: LOR, **(B)**: ROR, **(C)**: LOL, **(D)**: ROL.

Figure 5 showed that the GluCEST effect was positively correlated with Glu levels in four brain regions, including right optic radiation (ROR), left optic radiation (LOR), right occipital lobe (ROL) and left occipital lobe (LOL) in the acute group, with the R values of 0.554, 0.353, 0.596, 0.528, respectively. In the chronic group, the Gln levels in OR was positively correlated withTgAb (*F* = 0.909; *p* = 0.012), TPOAb (*F* = 0.873; *p* = 0.023), FT3 (*F* = 0.645; *p* = 0.032) and FT4 (*F* = 0.654; *p* = 0.029).

## Discussion

Whether there are metabolic and microstructural changes in intracranial visual pathways in mild TED patients has not been reported currently. Altogether, our present study has been the first report so far by using 1H-MRS, GluCEST and DKI techniques to demonstrate alterations in the metabolism and microstructure in intracranial visual pathways in mild TED patients before the onset of DON. We found that damage to visual pathways occurs in mild TED cases, offering new ideas for DON’s diagnosis (Liu et al., 2023) and being of impact for identifying early DON mechanisms, which is a gradual and continuous spatio-emporal progression.

### TED-associated metabolic changes by ^1^H-MRS and GluCEST

TCh levels were observed to increase by ^1^H-MRS in OR in the active and acute groups compared with HCs. It has been reported that the changes of Ch measured by MRS in glaucoma (Murphy et al., 2016; Sidek et al., 2016), and role of the cholinergic nervous system includes stimulating glutathione synthesis, preventing Glu excitotoxicity, protecting mitochondrial function to reduce oxidative damage and apoptosis (Faiq et al., 2019). Therefore, higher tCh levels in the active and acute groups may indicate a protective effect on improving the visual neuronal survival The changes of Ch may be related to the course of the disease. In our experiment, the level of Ch increased in the mild TED patients, but when the disease worsens in the later stage, it may be accompanied by undercompensation thus showing a decrease. Remarkably, their ROI was located in the occipital lobe, however, there was no statistical difference in Ch in the occipital lobe among all groups in our experiment. While in optic radiation, there was statistical difference in Ch, which may be related to the transmission order of the damage. The damage of the optic nerve was transmitted first to the optic radiation and then to the occipital lobe. Therefore, when studying early damage to the optic pathway, we could focus on changes in optic radiation, not just changes in the occipital lobe.

Compared with HC group, the amounts of Gln in OR in chronic group were significantly higher, together with a tendency increase of Glu, which might indicate chronic Glu-Gln cycle impairment in visual fibers over a TED course greater than 6 months. This Glu-Gln cycle sustains neural activity, and persistent disturbances of the Glu-Gln cycle flux may cause cognitive and affective symptoms in patients with Graves’ disease (Danielsen et al., 2008). Some other studies have suggested that visual processing depends on AMPA- and NMDA-type Glu receptors (Manookin et al., 2010). In patients with TED, increased intraorbital pressure may lead to impaired optic nerves. Therefore, the GluCEST effect in our study were significantly higher in the OR in the TED groups, indicating that increased Glu most likely represents damage to the OR and occipital lobe originating from the optic nerve (Kanaan et al., 2017). However, there was no significant difference in Glu levels between any two groups by ^1^H-MRS, although they showed a tendency increase in TED groups compared to HCs. Possible reason might be that the peak-overlap problem in 3.0 T 1H-MRS and partial volume effect reduce the accuracy of Glu quantification (Provencher, 1993). However, the GluCEST effect was significantly higher in TED groups and was positively correlated with Glu levels of the corresponding brain regions mentioned above, implying that GluCEST may be more sensitive than ^1^H-MRS to evaluate Glu levels, thanks for its high spatial-resolution imaging.

Correlation analysis of GD associated hormones and metabolites showed that changes of Gln levels in OR in chronic group were positively correlated with changes of serum TgAb, TPOAb, FT3, and FT4. Changes in serology may lead to changes in metabolites in visual pathways, which in true may indicate that the status of disease injury, which is a correlation with the dysregulation of the thyroid function, can be reflected by non-invasive detection of metabolites in the brain by ^1^H-MRS (Danielsen et al., 2008). However, the reasons behind this correlation are unclear. Whether serum hormones indirectly “communicate” with the brain through some mechanism and cause changes in brain metabolites remains to be further studied.

### TED related microstructural changes measured by DKI

In terms of microstructural changes assessed by DKI, the kurtosis parameters of MK, MD, FA, Ka and Kr can be used as imaging biomarkers for assessing microstructural damage (Gong et al., 2013). Our study showed that FA and Da were lower in the OR and Dr. was lower in the OL in the acute group, indicating alterations in demyelination and increased axonal membrane permeability in both crossing and non-crossing fibers (Lu et al., 2020; Sun et al., 2020). The decrease of FA value needs DWI data-sets into NODDI as a complement to further distinguish the neurite density and fiber distribution direction of the tissue alternations.MK acquired from DKI was exhibited to be sensitive to the microstructul changes of lesions, such as in breast cancer reported by Tang et al. (Huang et al., 2019; Tang et al., 2022). However, we did not find significant alterations of MK in TED patients while FA was shown to be changed markedly, suggesting that TED patients may be characterized by changes in nerve fibers rather than local microstructure alterations. Previous studies have also suggested that FA of the OR is associated with visual sensitivity in the optic pathway (de Blank et al., 2013). Thus, the microstructure of the visual pathway may be altered before the onset of DON and FA may be more sensitive than visual-evoked potentials as an objectively measured indicator.

DKI has been suggested to be sensitive for detecting alterations in diffusion, and our results differed from previous DTI studies in TED patients, which may be due to the methodological differences between DTI and DKI acquisition, processing parameters and the influence of the partial-volume effects (Falangola et al., 2013). In contrast to previous findings, here changes in intraorbital optic nerve fibers were only found in Ka in the active group, while FA and MD showed only a slight and insignificant decreasing trend, most likely due to the intersection of axons in the optic chiasm (Zeng et al., 2018) and the movement of the intraorbital optic nerves with eyeball movements (Staempfli et al., 2007; Engelhorn et al., 2012).

## Limitations

Our study had some limitations. First, the sample size in different TED groups was relatively small, thus using a larger sample with multiple TED types may yield a more accurate estimate. Second, this study was only limited to the cross-sectional design, which may bias our findings. Hence, continuous observation of the TED patients before and after treatment would be preferable. Third, correlation between the changes of serum hormones and brain metabolites is not clear, and the correlation between MRS and GluCEST may also have errors due to the size of ROI. Further study is needed to explore the association between imaging findings and hormones alterations.

## Conclusion

To conclude, our study was the first report so far by using ^1^H-MRS, GluCEST and DKI techniques to demonstrate alterations in the metabolism and microstructure in intracranial visual pathways in TED patients before the onset of disabling dysthyroid optic neuropathy. GluCEST is more sensitive than ^1^H-MRS to monitor the Glu alterations in visual pathway in TED patients. The level of Gln is closely related to associated hormones alterations, indicating that disease injury status can be reflected through non-invasive metabolites detection by brain ^1^H-MRS. FA is the most sensitive DKI index of visual pathway impairment in TED patients. We found that damage to visual pathways occurs in mild TED cases, which not only offer a new approach to the diagnosis of DON, but also demonstrates neuropathy in TED is a gradual and continuous spatio-emporal progression. However, a multicenter prospective study with a larger cohort should be performed in near future to validate these results.

## Data availability statement

The original contributions presented in the study are included in the article/supplementary materials, further inquiries can be directed to the corresponding author/s.

## Ethics statement

The studies involving humans were approved by the Ethics Committee of Second Affiliated Hospital of Shantou University Medical College. The studies were conducted in accordance with the local legislation and institutional requirements. The participants provided their written informed consent to participate in this study.

## Author contributions

LL: Conceptualization, Data curation, Investigation, Methodology, Software, Writing – original draft, Writing – review & editing. LZ: Data curation, Methodology, Writing – review & editing. HH: Methodology, Software, Writing – review & editing. JG: Conceptualization, Formal analysis, Investigation, Writing – review & editing. XZ: Data curation, Methodology, Software, Writing – review & editing. YL: Project administration, Resources, Writing – review & editing. RW: Conceptualization, Funding acquisition, Project administration, Supervision, Writing – review & editing.
